# Traitement des fractures des plateaux externes par vissage percutané assisté par arthroscopie

**DOI:** 10.11604/pamj.2015.21.287.6480

**Published:** 2015-08-18

**Authors:** Merouane Abouchane, Amine Belmoubarik, Hamza Benameur, Ahmed Reda Haddoun, Mohammed Nechad

**Affiliations:** 1 Service de Traumatologie Orthopédie Aile 4 CHU Ibn Rochd, Casablanca, Maroc

**Keywords:** plateau tibial, fracture, séparation, arthroscopie, tibial plate, fracture, separation, arthroscopy

## Abstract

Le but de notre étude est d'évaluer les résultats de fractures des plateaux tibiaux externes traitées par ostéosynthèse percutanée assistée par arthroscopie. Dix patients (8 hommes et 2 femmes) de 32 ans en moyenne ont subi cette intervention afin de réparer des fractures des plateaux tibiaux Schatzker I-III. Après avoir appliqué un garrot pneumatique, nous avons réduit et fixé la fracture au moyen de vis cannelées souschondrales. Lésions associées retrouvent deux lésions partielles du ménisque externe ont été retrouvé, traitées par résection partielle. Une orthèse de genou été de mise à but antalgique et protectrice pendant six semaines avec béquillage et interdiction de l'appui pour une durée de douze semaines avec reprise d'appui partiel au delà. La durée d'hospitalisation été d'une moyenne de cinq jours. La rééducation passive a été commence le lendemain de l'intervention et continuait dans chez un kinésithérapeute à la sortie du patient du service. Le suivi été à J7, J15, 1mois, 3mois, 6 mois puis tous les 6 mois. Neuf de nos patients ont été revu régulièrement sauf un perdu de vue. Le recul moyen de notre série été de 16 mois (10 et 24 mois). Le score de Lysholm a été utilisé pour évaluer les résultats cliniques chez nos neuf patients: excellent chez trois patients bons chez trois moyen chez un seul et mauvais chez deux patients. Tous nos neuf patients ont consolidé (figure 10 contrôle scopique d un article). Aucune gonarthrose n'a été note chez nos neuf patients due essentiellement au recul moyen faible de 16 mois. Le traitement des fractures des plateaux tibiaux externes assisté par arthroscopie produit des résultats satisfaisants et peut être accepté comme solution de rechange efficace au traitement des fractures des plateaux tibiaux causées par un choc de faible énergie.

## Introduction

Les fractures des plateaux tibiaux représentent 1% de toutes les fractures. Leur prise en charge demeure difficile. Il s'agit de fractures articulaires, nécessitant une réduction la plus anatomique possible, une ostéosynthèse stable permettant de débuter précocement la rééducation afin d'obtenir les meilleurs résultats fonctionnels [1, 2]. Un traitement non adapté peut aboutir à des séquelles susceptibles d'avoir un impact social important. La technique d'ostéosynthèse percutanée sous contrôle arthroscopique et fluoroscopue décrite initialement par Caspari et al. [3] et Jennings [4] a trouvé sa place pour les fractures de type I à III selon la classification de Schatzker [3, 5]. Elle a pour avantage théorique d'être une technique mini-invasive, avec une morbidité moindre, notamment en termes de dévascularisation, de permettre un contrôle de la réduction de la fracture et de réaliser un bilan des lésions associées, ainsi que leurs traitements [6]. La littérature récente concernant la prise en charge arthroscopique de ces fractures rapporte de bons résultats fonctionnels et radiologiques à court terme [3, 5, 7, 8].

## Méthodes

**Figure 1:**
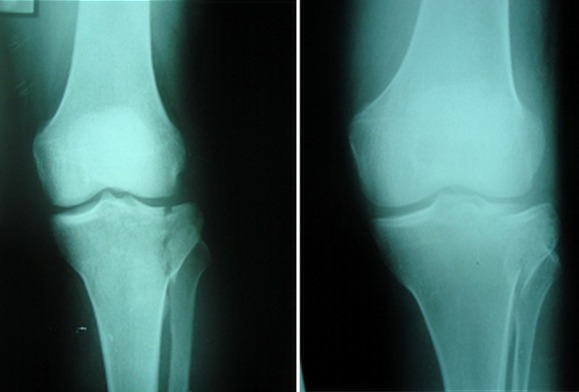
Fracture séparation pure

**Figure 2:**
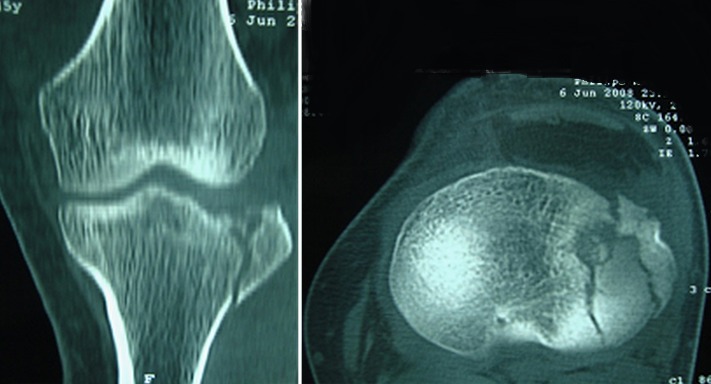
Fracture séparation enfoncement

**Figure 3:**
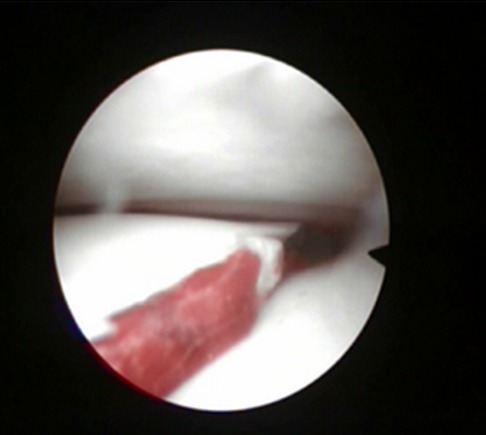
Vue arthroscopique d'une séparation pure

**Figure 4:**
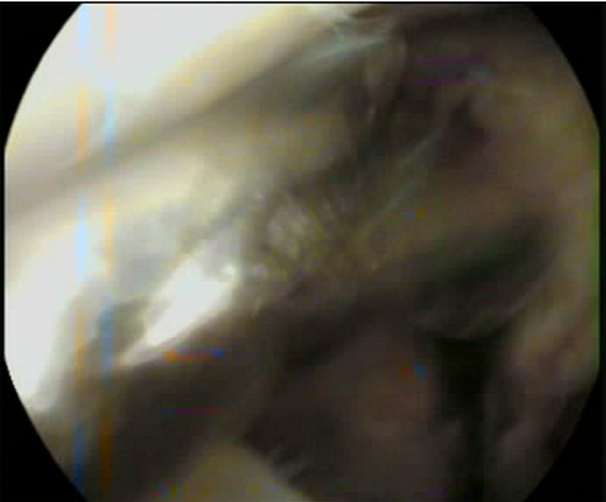
Vue endoscopique d'un enfoncement

**Figure 5:**
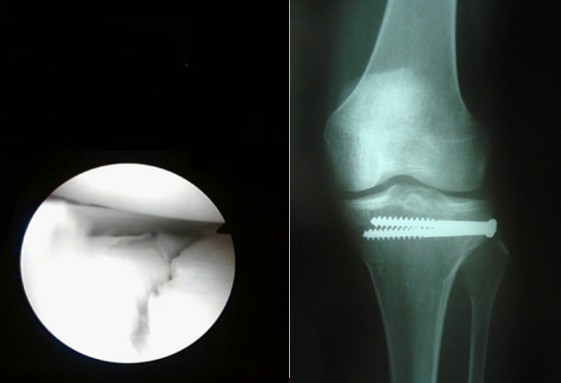
A: vue arthroscopique de réduction de séparation pure; B: radiographie de controle après vissage

**Figure 6:**
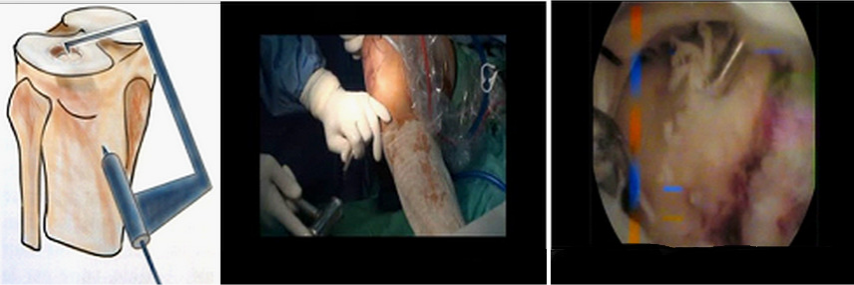
Technique de relèvement de l'enfoncement

**Figure 7:**
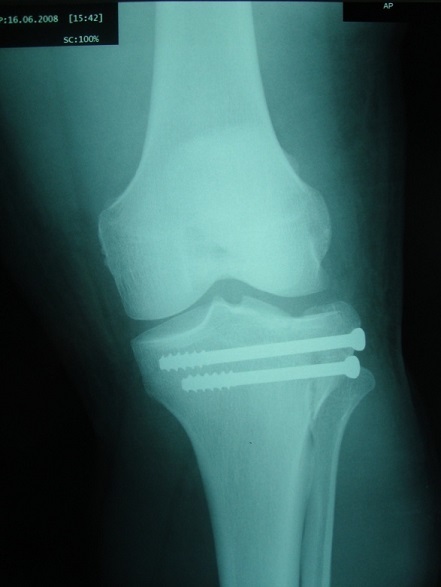
Radiographie de contrôle

Entre 2012 et 2014 1 dix patients présentant une fracture du plateau tibial externe déplacée (Schatzker I à III) ont été traité dans notre structure sous assistance arthroscopique, bénéficiant tous d'une fixation interne. Huit patients été des hommes et deux été des femmes, avec une moyenne d'âge de 32 ans (19 et 55 ans). On note huit AVP, un accident de sport et une chute. La durée entre l'hospitalisation et le traitement chirurgical été d'une moyenne de sept jours (bilan préopératoire et disponibilité du matériel). Le bilan préopératoire inclut radiographie du genou de face, de profile et une incidence de ¾, la TDM du genou concerné avec reconstruction 2D et 3D été réalisée chez 6 patients à chaque fois qu'on suspecte un enfoncement associe. Au terme de ce bilan: cinq patients présentent une fracture séparation pure (type I) (Figure 1), trois patients avec une fracture séparation enfoncement (type II) (Figure 2) et deux patients avec un enfoncement pure (type III). L'acte opératoire s'est déroule sous anesthésie générale, décubitus dorsal, un garrot à la racine du membre été mis en place avec préparation systématique de la crête iliaque homolatérale et un arthrostress été utilisé pour toutes les interventions. L'arthroscope été introduit par voie anteroexterne et un lavage abondant été réalisé avant de commencer l'exploration de l'articulation. Le bilan articulaire retrouve: Fracture: cinq patients avec fracture séparation pure (Figure 3) et cinq avec une composante d'enfoncement (Figure 4) dont trois mixtes rejoignant le bilan préopératoire radiologique réalisé. Lésions associées: deux lésions partielles du ménisque externe ont été retrouvées, traitées par résection partielle. Pour les fractures séparation pure: un vissage par une vis (une fois) deux vis (deux fois) ou trois vis spongieuses 6,5 mm (deux fois) été satisfaisant (Figure 5). Pour les fractures avec enfoncement: le relèvement de l'enfoncement été réalisé à l'aide de viseur de ligamentoplastie avec mise en place d'une broche guide (Figure 6), des chasses greffons de calibre convenable introduits a travers la fracture pour les fracture mixtes et après un fenêtrage de la corticale pour les fracture enfoncement pure ont pu relever l'enfoncement. La réduction été suivie sous contrôle fluoroarthroscopique. La greffe été nécessaire chez un seul patient par un greffon iliaque. La fixation été réalisée par deux ou trois vis spongieuses 6,5 mm (Figure 7). Le drainage été systematique chez tous nos patients avec ablation du drain le 2 ou 3 eme jour. Une orthèse de genou été de mise à but antalgique et protectrice pendant six semaines avec béquillage et interdiction de l'appui pour une durée de douze semaines avec reprise d'appui partiel au delà. La durée d'hospitalisation été d'une moyenne de cinq jours. La rééducation passive a été commence le lendemain de l'intervention et continuait dans chez un kinésithérapeute à la sortie du patient du service. Aucune complications immédiate n'a été note à part un syndrome douloureux post opératoire régional sans signe neurovasculaires chez deux patients, ayant cédé sous traitement médical et surélévation du membre. Le suivi été à J7, J15, 1mois ,3mois, 6 mois puis tous les 6 mois. Neuf de nos patients ont été revu régulièrement sauf un perdu de vue. Le recul moyen de notre série été de 16 mois (10 et 24 mois).

## Résultats

**Résultats cliniques:** la stabilité du genou été évaluée retrouvant: quatre cas de laxité: avec un Lachman positif chez deux patients, un valgus exagéré chez un seul patient et un avec un Lachman et valgus exagéré. Une raideur articulaire été note chez deux patients dont une mobilisation sous AG été prescrite chez un seul et la continuation de la rééducation chez l'autre permettant un secteur de mobilité entre 90 et 120°. Le score de Lysholm a été utilisé pour évaluer les résultats cliniques chez nos neuf patients: excellent chez trois patients bons chez trois, moyen chez un seul et mauvais chez deux patients.

**Résultats radiologiques:** tous nos neuf patients ont consolidé. Deux cas de tassement de 1 à 2 mm été noté sans indication à une reprise. Aucune gonarthrose n'a été note chez nos neuf patients due essentiellement au recul moyen faible de 16 mois. La goniométrie n'a pas été réalisée.

## Discussion

La complication majeure des fractures de l'extrémité proximale du tibia est l'arthrose, exigeant ainsi une réduction anatomique de la surface articulaire, la restauration de l'alignement axial et une fixation stable, permettant une mobilisation active et passive immédiate pour obtenir des résultats satisfaisants [9]. Quel qu'il soit le traitement, le but ultime est de préserver les amplitudes articulaires du genou [2, 10, 11]. En général, le traitement chirurgical est indiqué pour les fractures articulaires avec un enfoncement et/ou séparation de 3 à 5 mm et instabilité en varus/valgus plus de 10° [12, 13]. Le traitement chirurgical habituel, qui comprend une réduction à foyer ouvert avec fixation interne, a démontré des résultats satisfaisants mais avec une dissection extensive [14, 15]. En plus cette technique dans plusieurs situations nécessite une arthrotomie sous méniscale avec relèvement méniscal pour un meilleur controle de la réduction articulaire, ce qui est souvent source de raideur importante, douleur prolongée et problèmes de cicatrisation [5, 13, 15]. La fixation percutanée arthroscopiquement assistée, recommandée pour la première fois par Caspari [3] et Jennings [16], est devenu populaire depuis son utilisation comme moyen diagnostique. Cette technique permet une visualisation directe du trait articulaire, une meilleure réduction, moins de morbidité par rapport la technique à ciel ouvert et un traitement immédiat des lésions intra-articulaires associées, la prévention des complications des parties molles et la possibilité de lavage articulaire comprenant les débits chondraux et l'hématome [17–20]. Fowble et al [5] rapporte que les résultats du traitement sous arthroscopie sont meilleurs par rapport à la technique à ciel ouvert, et met en évidence une bonne réduction anatomique, un taux de complications moindre et un délai plus court pour la reprise de l'appui. Ohdera et al [21] ne rapportent pas de différence concernant la durée de l'acte chirurgical, les amplitudes articulaires du genou et les résultats cliniques entre les patients traite opérés a ciel ouvert et ceux opérés sous arthroscopie; cependant ils notent une rééducation plus rapide et plus facile parmi les patients traites sous arthroscopie. Il est rapporté que ce n'est pas toutes les fractures des plateaux tibiaux sont accessibles au traitement sous arthroscopie, ainsi les fractures classées Schatzker IV et V, dues à des traumatismes à haute energie, associées souvent à des risques de syndrome de loges ne présentent pas une bonne indication [2, 12, 19]. Par ailleurs les type I, II et III représentent les meilleures indications pour cette technique selon Tornetta et Kayali [9, 19].

## Conclusion

L'ostéosynthèse percutanée sous contrôle arthroscopique est le traitement de choix pour les fractures du plateau tibial de types I à III dans la classification de Schatzker. Elle a pour avantages d'avoir une morbidité plus faible, de contrôler la réduction et de permettre un bilan des lésions associées ainsi que leurs traitements. Au vu de notre étude, réalisé sur un petit échantillon avec un faible recul, ne permet pas, malheureusement, de confirmer les résultats des séries à moyen terme et à long terme, qui démontrent les avantages de l'assistance arthroscopique dans ce genre de fractures puisque les résultats cliniques et fonctionnels sont satisfaisants et ne se dégradent pas dans le temps par rapport aux séries à court terme. Ceci nous incite à élargir le champ de l'étude avec un suivi à moyen et long terme.

## References

[1] Cassard X, Beaufils P, Blin JL, Hardy P (1999). Osteosynthesis under athroscopic control of separated tibial plateau fractures, 26 case reports. Rev Chir Orthop Reparatrice Appar Mot..

[2] Guanche CA, Markman AW (1993). Arthroscopic management of tibial plateau fractures. Arthroscopy..

[3] Caspari RB, Hutton PM, Whipple TL, Meyers JF (1985). The role of arthroscopy in the management of tibial plateau fractures. Arthroscopy..

[4] Jennings JE (1985). Arthroscopic management of tibial plateau fractures. Arthroscopy..

[5] Fowble CD, Zimmer JW, Schepsis AA (1993). The role of arthroscopy in the assessment and treatment of tibial plateau fractures. Arthroscopy..

[6] Hannouche DD, Duparc F, Beaufils P (2006). The arterial vascularization of the lateral tibial condyle: anatomy and surgical applications. Surg Radiol Anat..

[7] Asik M, Cetik O, Talu U, Sozen YV (2002). Arthroscopy-assisted operative management of tibial plateau fractures. Knee Surg Sports Traumatol Arthrosc..

[8] Roerdink WH, Oskam J, Vierhout PA (2001). Arthroscopically assisted osteosynthesis of tibial plateau fractures in patients older than 55 years. Arthroscopy..

[9] Kayali C, Oztürk H, Altay T, Reisoglu A, Agus H (2008). Arthroscopically assisted percutaneous osteosynthesis of lateral tibial plateau fractures. Can J Surg..

[10] Houben PFJ, Linden ES, Wildenberg FAJM (1997). Functional and radiological outcome after intra-articular tibial plateau fractures. Injury..

[11] Scheerlinck T, Ng CS, Handelberg F (1998). Medium-term results of percutaneous, arthroscopically-assisted osteosynthesis of fractures of the tibial plateau. J Bone Joint Surg Br..

[12] McClellan RT, Comstock CP (1999). Evaluation and treatment of tibial plateau fractures. Curr Opin Orthop..

[13] Hung SS, Chao EK, Chan YS (2003). Arthroscopically assisted osteosynthesis for tibial plateau fractures. J Trauma..

[14] Karas EH, Weiner LS, Yang EC (1996). The use of an anterior incision of the meniscus for exposure of tibial plateau fractures requiring open reduction and internal fixation. J Orthop Trauma..

[15] Stevens DG, Beharry R, McKee MD (2001). The long-term functional outcome of operatively treated tibial plateau fractures. J Orthop Trauma..

[16] Jennings JE (1985). Arthroscopic management of tibial plateau fractures. Arthroscopy..

[17] Ruth JT (2001). Fractures of the tibial plateau. Am J Knee Surg..

[18] Van Trommel MF, Simonian PT, Potter HG (1998). Arthroscopically-aided lateral meniscal repair and reduction of lateral tibial plateau fracture: long-term follow up with MR imaging. Knee..

[19] Tornetta P (2002). Arthroscopic elevation with grafting. J Orthop Trauma..

[20] Muezzinoglu US, Guner G, Gurfidan E (1995). Arthroscopixally assisted tibial plateau fracture management: a modified method. Arthroscopy..

[21] Ohdera T, Tokunaga M, Hiroshima S (2003). Arthroscopic management tibial plateau fractures-comparison with open reduction method. Arch Orthop Trauma Surg..

